# Current status of collaborative relationships between dialysis facilities and dental facilities in Japan: results of a nationwide survey

**DOI:** 10.1186/s12882-015-0001-0

**Published:** 2015-02-12

**Authors:** Masami Yoshioka, Yasuhiko Shirayama, Issei Imoto, Daisuke Hinode, Shizuko Yanagisawa, Yuko Takeuchi

**Affiliations:** Department of Oral Health Science and Social Welfare, Institute of Health Biosciences, The University of Tokushima Graduate School, Tokushima, Japan; Department of Community Medicine and Welfare, Institute of Health Biosciences, The University of Tokushima Graduate School, Tokushima, Japan; Department of Human Genetics, Institute of Health Biosciences, The University of Tokushima Graduate School, Tokushima, Japan; Department of Hygiene and Oral Health Science, Institute of Health Biosciences, The University of Tokushima Graduate School, Tokushima, Japan

**Keywords:** Oral health care, Hemodialysis patient, Collaborative relationship between medical and dental facilities, Nationwide questionnaire

## Abstract

**Background:**

Recent studies have reported an association between periodontal disease and mortality among dialysis patients. Therefore, preventive dental care should be considered very important for this population. In Japan, no systematic education has been undertaken regarding the importance of preventive dental care for hemodialysis patients—even though these individuals tend to have oral and dental problems. The aim of this study was to investigate the current state of collaborative relationships between hemodialysis facilities and dental services in Japan and also to identify strategies to encourage preventive dental visits among hemodialysis outpatients.

**Methods:**

A nationwide questionnaire on the collaborative relationship between dialysis facilities and dental facilities was sent by mail to all medical facilities in Japan offering outpatient hemodialysis treatment.

**Results:**

Responses were obtained from 1414 of 4014 facilities (35.2%). Among the 1414 facilities, 272 (19.2%) had a dental service department. Approximately 100,000 dialysis outpatients were receiving treatment at these participating facilities, which amounts to one-third of all dialysis patients in Japan. Of those patients, 82.9% received hemodialysis at medical facilities without dental departments. Only 87 of 454 small clinics without in-house dental departments (19.2%) had collaborative registered dental clinics. Medical facilities with registered dental clinics demonstrated a significantly more proactive attitude to routine collaboration on dental matters than facilities lacking such clinics.

**Conclusions:**

Our nationwide survey revealed that most dialysis facilities in Japan have neither an in-house dental department nor a collaborative relationship with a registered dental clinic. Registration of dental clinics appears to promote collaboration with dental facilities on a routine basis, which would be beneficial for oral health management in hemodialysis patients.

**Electronic supplementary material:**

The online version of this article (doi:10.1186/s12882-015-0001-0) contains supplementary material, which is available to authorized users.

## Background

The close association between oral health and systemic disease has been elucidated in recent decades [1,2]. Dialysis patients tend to have oral health problems, which are related to several factors, including restriction of oral fluid intake and relative immunosuppression [3,4]. Oral and dental diseases are a source of active infection in individuals with renal disease, and they can compromise general health and impede medical management of dialysis patients. Indeed, a possible association between periodontal status and mortality has been reported among dialysis patients [5]. Periodontal disease is associated with cardiovascular disease and is thought to accelerate systemic atherosclerosis [6]. Kshirsagar *et al*. reported a strong association between moderate to severe periodontal disease and cardiovascular disease mortality among hemodialysis patients [7]. Chen *et al*. identified an association between periodontitis and increased risk of death among patients undergoing long-term hemodialysis [8]. Both reports suggested the need for larger studies to confirm this association [7,8]. A multinational prospective longitudinal observational large-cohort study on oral diseases in hemodialysis patients is currently ongoing [9]. Because dialysis patients need more professional oral health care than other patients, the dental professional is a fundamental provider in their overall management.

In Japan, most dental professionals work in private dental clinics: only 12.2% of dentists and 4.7% of dental hygienists are employed in hospitals [10,11]. Therefore, most medical practitioners must refer patients with oral problems to dental facilities outside their hospitals or clinics. Because hemodialysis patients need to spend 3 days a week receiving dialysis treatment, patients undergoing treatment at hospitals or clinics that do not provide dentistry may often miss preventive dental visits.

In this study, we investigated the proportion of dialysis facilities in Japan with dental departments for providing professional oral health-care services. In addition, we evaluated the current state of collaborative relationships between dialysis hospitals or clinics and dental facilities outside dialysis facilities. Our aim was to determine strategies to raise awareness of the need for basic preventive dental care among dialysis patients and to encourage such patients to undertake holistic oral health care.

## Methods

All hospitals and clinics in Japan listed on their prefecture’s web site as having a dialysis facility in June 2013 were included in this study. A mailing list of those facilities was purchased from a research service company (Tokyo Data System Co. Ltd., Saitama, Japan). Questionnaires (Additional file 1) were mailed to the individual in charge of the dialysis section at each facility. In this study, written informed consent for participation was not obtained from the participants: we regarded the reply as signifying agreement to participate—as explained in the document that we sent together with the questionnaire to each facility. We divided the responding facilities into three groups and eight subgroups according to the number of hospital beds: small clinics (0 or 1–19 beds); small hospitals (20–100 or 101–200 beds); and large hospitals (201–300, 301–400, 401–500, or >500 beds). The questionnaire consisted of 12 questions, including the following: “Does your facility have a dental or oral surgery department?”; “How many dental professionals does your facility employ?”; “Do you collaborate with dental facilities?”; and “Do you agree with measures to promote dental visits among dialysis patients?”

The survey responses were coded and analyzed using the SPSS 17.00 statistical package (SPSS Japan Inc., Tokyo, Japan). A chi-square test was used to compare answers among the groups. Statistical significance was accepted at a level of 0.05 and lower.

This study was approved by the ethics committee of Tokushima University Hospital (No. 1743).

## Results

### Distribution of dialysis facilities and patients

**Figure 1:**
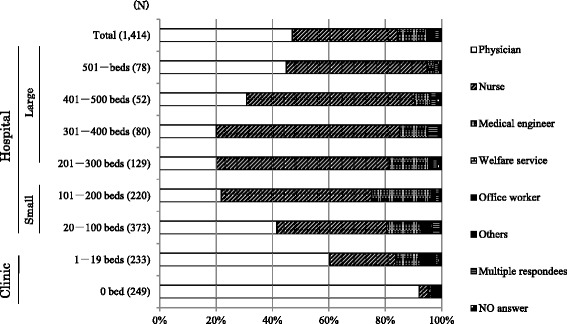
**Distribution of professionals completing the questionnaire, by facility size.** N, number of facilities.

Valid responses were obtained from 1414 of 4014 dialysis facilities (35.2%). The distribution of professionals who completed the questionnaire is shown in Figure 1. At small clinics, physicians generally completed the questionnaire; at large hospitals, the questionnaire was usually answered by nurses. The proportion of dialysis facilities with and without associated dental departments according to facility size is shown in Table 1. The percentage of dialysis patients receiving treatment at facilities with and without dental departments according to facility size appears in Table 2. Only 272 of the 1414 facilities with valid responses (19.2%) had a dental department; less than 10% of the small clinics had a dental department. Based on the survey responses, an estimated 100,000 dialysis outpatients received treatment at the participating facilities, which amounts to one-third of dialysis patients in Japan [12]. Of those patients, 82.9% visited medical facilities without dental departments (Table 2).

**Table 1:** **Proportion of surveyed dialysis facilities with and without dental departments**

**Number of beds**	**Facilities without dental department**		**Facilities with dental department**		**Total**	
Small Clinic						
0	245	(98.4%)	4	(1.6%)	249	(100%)
1-19	218	(93.6%)	15	(6.4%)	233	(100%)
Small Hospital						
20-100	335	(89.8%)	38	(10.2%)	373	(100%)
101-200	173	(78.6%)	47	(21.4%)	220	(100%)
Large Hospital						
201-300	88	(68.2%)	41	(31.8%)	129	(100%)
301-400	44	(55.0%)	36	(45.0%)	80	(100%)
401-500	24	(46.2%)	28	(53.8%)	52	(100%)
>500	15	(19.2%)	63	(80.8%)	78	(100%)
Total	1142	(80.8%)	272	(19.2%)	1414	(100%)

**Table 2:** **Percentage of dialysis patients** ^**a**^ **treated at facilities with and without dental departments**

**Number of beds**	**Facilities without dental department**		**Facilities with dental department**		**Total**	
Small Clinic						
0	21,154	(98.5%)	325	(1.5%)	21,479	(100%)
1-19	14,339	(96.8%)	479	(3.2%)	14,818	(100%)
Small Hospital						
20-100	28,911	(91.8%)	2587	(8.2%)	31,498	(100%)
101-200	9874	(73.1%)	3625	(26.9%)	13,499	(100%)
Large Hospital						
201-300	5129	(66.2%)	2621	(33.8%)	7750	(100%)
301-400	3334	(54.0%)	2839	(46.0%)	6173	(100%)
401-500	1817	(50.3%)	1794	(49.7%)	3611	(100%)
>500	821	(19.5%)	3399	(80.5%)	4220	(100%)
Total	85,379	(82.9%)	17,669	(17.1%)	103,048	(100%)

^a^The number of patients in each group was estimated.

### Employment of dental professionals

We also investigated the employment of dental professionals (dentists or dental hygienists) at dialysis facilities. Dental professionals were employed at 90.4% of dialysis facilities with dental departments and at 3.2% of dialysis facilities without dental departments. Although dental professionals were employed at most facilities with dental departments, the average number of dental professionals at those facilities was very small.

### Current collaborative relationships between dialysis and dental facilities and future prospects

**Figure 2:**
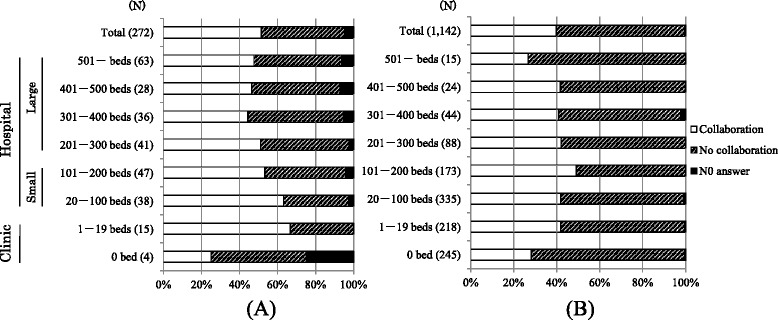
**Routine collaboration with dental facilities among dialysis hospitals and clinics. (A)** with dental departments, **(B)** without dental departments; N, number of facilities.

Routine collaboration with dental facilities was relatively rare among facilities without dental departments compared with those having dental departments (Figure 2). Most routine collaborations with dental facilities were referral for oral prophylaxis and/or dental treatment and consultation (29.4 and 24.5%, respectively).

We also examined the prospects for future collaboration with dental facilities: 14 of 272 facilities with dental departments (5.1%), and 216 of 1142 without dental departments (18.9%) replied that collaboration was unnecessary. Of 463 small clinics without dental departments, 115 (24.8%) replied that collaboration was not necessary.

**Figure 3:**
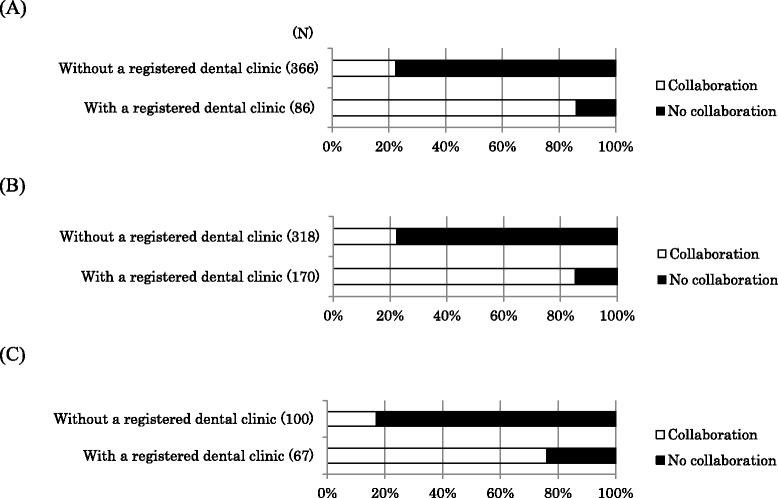
**Routine dental collaboration for hemodialysis facilities with and without a registered dental clinic. (A)** Dialysis clinics (0-19 beds) without dental departments, **(B)** Dialysis hospitals (20-200 beds) without dental departments **(C)** Dialysis hospitals (>200 beds) without dental departments; N, number of facilities.

**Figure 4:**
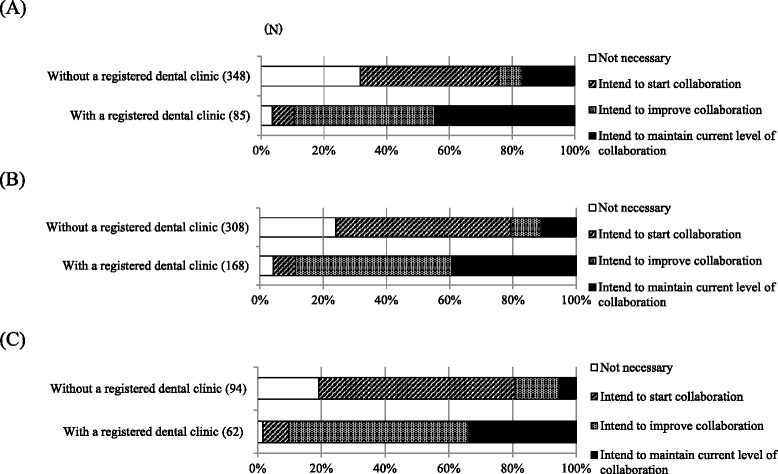
**Attitude towards dental collaboration for facilities with and without a registered dental clinic. (A)** Dialysis clinics (0-19 beds) without dental departments, **(B)** Dialysis hospitals (20-200 beds) without dental departments **(C)** Dialysis hospitals (>200 beds) without dental departments; N, number of facilities.

Next, we divided the facilities without dental departments into two groups: facilities with a collaborative registered dental clinic and those without a registered dental clinic. As shown in Table 3, 80.8% of small dialysis clinics did not have a registered dental clinic. The percentage of facilities having a registered dental clinic was lower among clinics than hospitals (Table 3). A higher proportion of medical facilities with a registered dental clinic reported routine collaboration with a dental facility than those not having a registered dental clinic (*p* <0.01, chi-square test; Figure 3). Among facilities with a registered dental clinic, 83.6% replied that they collaborated routinely with a dental facility. Medical facilities with a registered dental clinic also demonstrated a more positive attitude toward collaboration with dental facilities than facilities without such clinics (Figure 4).

**Table 3:** **Percentage of facilities without dental departments having a collaborating registered dental clinic**

**Size of facility**	**Having a registered dental clinic**		**Lacking a registered dental clinic**	
Small Clinic				
(0–19 beds)	87	(19.2%)	367	(80.8%)
Small Hospital				
(20–200 beds)	170	(34.7%)	320	(65.3%)
Large Hospital				
(>200 beds)	67	(39.9%)	101	(60.1%)
Total	324	(29.1%)	788	(70.9%)

**Figure 5:**
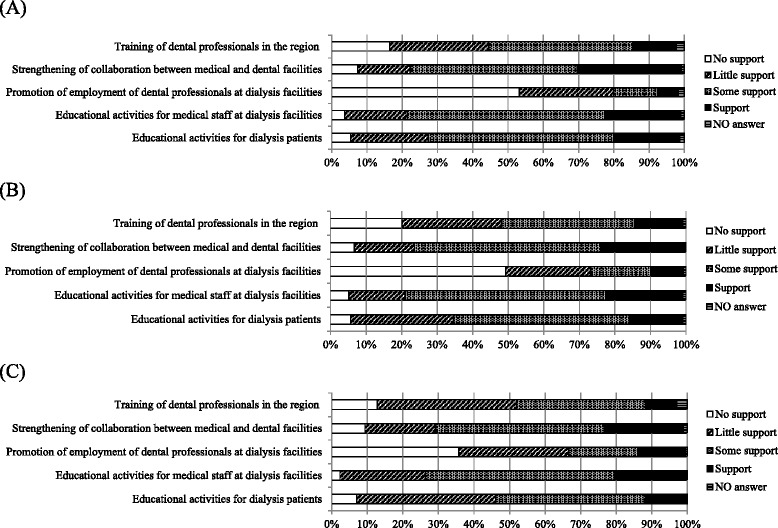
**Support for five measures to promote dental visits among dialysis patients. (A)** Dialysis clinics (0-19 beds) without dental departments, **(B)** Dialysis hospitals (20-200 beds) without dental departments **(C)** Dialysis hospitals (>200 beds) without dental departments.

We asked respondents to indicate their degree of support for five measures to encourage dental visits among dialysis patients (Question 10 in Additional file 1). In all groups, “educational activities for medical staff at hemodialysis facilities” had the highest support; that was followed by “strengthening the collaboration between medical and dental facilities” and “educational activities for patients.” In contrast, “promoting the employment of dental professionals at hemodialysis facilities” received less support than expected (Figure 5). Among small clinics, “promoting the employment of dental professionals at hemodialysis facilities” had relatively low support; however, “educational activities for patients” had relatively high support compared with that expressed by hospital groups (Figure 5).

## Discussion

Our nationwide survey revealed that most dialysis patients in Japan receive hemodialysis therapy at medical facilities without dental departments. Therefore, dialysis patients who need oral prophylaxis or dental treatment have to visit external dental facilities. In the dental treatment of dialysis patients, information about the patient’s general medical condition and current medications, such as anticoagulants, is essential. A collaborative relationship between the physician and dentist is therefore indispensable.

Collaborative interdisciplinary medical care for cancer patients (including relationships among medical and dental professionals) is currently being promoted in Japan, although this is a recent development. Dental professionals are distinct from physicians, and there is a separate undergraduate curriculum for each in both Japan and Hong Kong [13]. To provide holistic health care, more intensive collaboration between medical and dental professionals should be encouraged [13].

In Japan, dental professionals treating dialysis patients mainly focus on susceptibility to infection and bleeding during oral surgery. Dialysis patients are seldom advised about oral health by physicians because preventive dental care is not a primary consideration for most medical professionals. A similar situation has been reported in Brazil, where nephrologists and nurses working with chronic kidney disease patients demonstrated good general knowledge about periodontal disease, but they had a low rate of referral for specialist treatment [14].

Preventive dental care visits remain relatively uncommon in Japan; this is because universal health insurance covers basic curative dental care, whereas its coverage of preventive dental care is limited [15]. It has been reported that dental visits in the public health-care setting in the United States are extremely infrequent, particularly among patients with chronic kidney disease [16]. Dialysis providers seldom advise their patients about oral health because preventive dental care is not their primary consideration. Further study is needed to clarify the barriers to successfully providing dialysis patients with preventive dental care. Through their close relationship to dialysis patients, the medical staff at dialysis facilities could serve as effective advisors in promoting preventive dental care. Education about the importance of preventive dental care should be provided for both medical staff at dialysis facilities and patients.

We proposed five measures to encourage routine dental care among dialysis patients and evaluated the degree of support for each measure. We found that the feasibility of employing dental professionals at dialysis facilities was very low. Since only a few dental professionals are employed at hospitals and clinics in Japan—even at facilities with dental departments—we believe that promoting the employment of dental professionals at dialysis facilities would be a major challenge. By contrast, we found relatively strong support for educational activities for medical staff at dialysis facilities, educational activities for patients, and reinforcing the collaboration between medical and dental facilities. Dialysis hospitals and clinics with a collaborating registered dental clinic tended to maintain routine relationships with the dental facility, and they were more positive toward collaboration with dental facilities. Therefore, holistic oral health management could be promoted by encouraging dialysis hospitals and clinics without dental departments to register a collaborating dental clinic and to enhance routine collaboration with dental professionals.

Because this was a cross-sectional survey, we cannot conclude that having a collaborating dental clinic leads to routine collaboration with a dental facility, or vice versa. However, we found that almost all facilities with a collaborating dental clinic were more proactive toward routine collaboration with dental clinics—even if they had not yet initiated such collaboration. Medical facilities in Japan are not required to work with a collaborative dental clinic. Having a collaborative dental clinic may be a first step in promoting the dental health management of dialysis patients and increasing the opportunities for collaboration with dental professionals.

This survey had an overall response rate of 35.2%. However, each group showed a different response rate: 26.1% for small clinics; 52.5% for small hospitals; and 29.4% for large hospitals. Owing to the relatively large difference in response rates among the three groups, we calculated the responses for each group and subgroup, and we limited the statistical analyses to within the same group.

According to a 2011 national survey of medical facilities, 1.6% of medical clinics (0–19 beds) and 14.4% of hospitals (>19 beds) included a dental department [17]. In the present study, 3.9% of small clinics and 27.1% of hospitals had a dental department. This difference indicates that facilities without dental departments were less likely to participate in the present survey. Our results demonstrate that facilities without dental departments were less liable to have routine collaboration with a dental facility than facilities having dental departments. We need to consider novel strategies to promote collaborative relationships between dental facilities and medical facilities without dental departments.

In this study, we did not have any information on the outcomes of dialysis patients who received dental care as opposed to those who did not. However, the literature supports a bidirectional relationship between chronic kidney disease and periodontal disease [18]. Treating periodontal disease could decrease the systemic inflammatory burden—as indicated by C-reactive protein and IL-6 levels [18]. One study found that conventional periodontal therapy decreased C-reactive protein and increased hemoglobin levels in hemodialysis patients [19]. Preventive dental care could effectively suppress the systemic inflammatory burden of dialysis patients.

## Conclusions

Our nationwide survey on collaboration between hemodialysis and dental facilities revealed that most hemodialysis patients in Japan receive treatment at medical facilities without dental departments. Having a registered dental clinic was associated with a positive attitude toward collaboration with dental facilities. Therefore, we propose that routine collaboration between dialysis facilities and dental facilities may be promoted by using a registered dental clinic and that such collaboration can promote holistic health care for dialysis patients.

## Additional file

Additional file 1:
**Questionnaire.**
