# Supplementary material for: Current status of collaborative relationships between dialysis facilities and dental facilities in Japan: results of a nationwide survey
Source: BMC Nephrol. 2015 Feb 12;16:17. doi: 10.1186/s12882-015-0001-0 (PMC4358784; doi:10.1186/s12882-015-0001-0)
Supplement: Additional file 1: — Questionnaire. [file 12882_2015_1_MOESM1_ESM.pdf]

Questionnaire

1. What is your medical specialty?
(1) Physician (2) Nurse (3) Medical engineer (4) Welfare service
(5) Office worker (6) Other ( )

2. How many beds does your clinic/hospital have?
_____ beds

3. Which of the following clinical departments are present at your clinic/hospital? (multiple choices allowed)
(1) Internal medicine (2) Surgery (3) Obstetrics/gynecology (4) Ophthalmology
(5) Otorhinolaryngology (6) Urology (7) Dermatology (8) Pediatrics (9) Radiology
(10) Anesthesiology (11) Psychiatry/neurology (12) Plastic surgery (13) Orthopedics
(14) Neurosurgery

4. Does your facility provide hemodialysis on an outpatient basis?
(1) Yes (2) No
If "Yes," how many outpatients receive hemodialysis treatment at your facility?
__________ Patients

5. How many dentists/dental hygienists does your facility employ? (Please convert part-time employees to full-time equivalents.)
Dentists __________ Dental hygienists __________

6. What do you think the prospects are for the future employment of dental professionals at your facility?
(1) We don't have any plans to employ dental professionals in the future.
(2) We would like to consider employing dental professionals.
(3) We would like to consider increasing our current employment of dental professionals.

7. Does your facility have a dental/oral surgery department?
(1) Yes (2) No

8. If you answered "No" to question 7, do you have a collaborative registered dental facility?
(1) Yes (2) No

9. What do you think of collaborative relationships with dental facilities? (single choice)
(1) We think that they are unnecessary.
(2) We would like to initiate a collaborative relationship.
(3) We would like to improve our present collaborative relationship.
(4) We would like to maintain our present collaborative relationship because it is sufficient.

10. We are planning the following measures to promote preventive dental visits among hemodialysis patients. How much would you agree with each measure?

<Measure 1: Educational activities for patients>
(1) Disagree (2) Somewhat disagree (3) Somewhat agree (4) Agree

<Measure 2: Educational activities for medical staff at hemodialysis facilities>
(1) Disagree (2) Somewhat disagree (3) Somewhat agree (4) Agree

<Measure 3: Promoting employment of dental professionals at hemodialysis facilities>
(1) Disagree (2) Somewhat disagree (3) Somewhat agree (4) Agree

<Measure 4: Strengthening the collaboration between medical and dental facilities>
(1) Disagree (2) Somewhat disagree (3) Somewhat agree (4) Agree

<Measure 5: Training of dental professionals in the region>
(1) Disagree (2) Somewhat disagree (3) Somewhat agree (4) Agree

11. Do you routinely collaborate with a dental clinic/hospital?
(1) Yes (2) No

12. If you answered "Yes" to question 11, please indicate the type of routine collaboration currently established with the dental facility. (multiple choices allowed)
(1) Consultations for each patient
(2) Referral for dental treatment and oral prophylaxis
(3) Patient instruction/education
(4) Interactive conferences
(5) Training sessions
(6) Other activities ( )
